# Editorial: AI-powered insights: predicting treatment response and prognosis in breast cancer

**DOI:** 10.3389/fonc.2026.1996821

**Published:** 2026-10-29

**Authors:** Tamer Ahmed Farrag, Ashraf Elbatanony, Mona Zaky, Khaled Abdelwahab, Yazan Masannat, Mohamed Shehata, Mostafa A. Elhosseini

**Affiliations:** 1 Department of Electrical Engineering, College of Engineering, Taif University, Taif, Saudi Arabia; 2 Department of Cariology, Faculty of Medicine, Al Azhar University, Cairo, Egypt; 3 Diagnostic Radiology Department, Faculty of Medicine, Mansoura University, Mansoura, Egypt; 4 Surgical Oncology Department, Mansoura Oncology Center, Mansoura University, Mansoura, Egypt; 5 Bloomfield Hospital, Mid and South Essex NHS Foundation Trust, Basildon, United Kingdom; 6 Computer Science Major, James C. Bowling School of Business, Midway University, Midway, KY, United States; 7 Computers and Control Systems Engineering Department, Mansoura University, Faculty of Engineering, Mansoura, Egypt; 8 Department of Information Systems, College of Computer Science and Engineering, Taibah University, Yanbu, Saudi Arabia

**Keywords:** artificial intelligence, breast cancer, computational oncology, machine learning, multi-omics, pathomics, precision medicine, radiomics

## 1 Introduction

Breast cancer remains a leading cause of cancer-related mortality among women worldwide, necessitating continuous refinement of clinical management strategies. Historically, diagnostic paradigms have relied heavily on traditional imaging modalities, such as mammography, ultrasound, and magnetic resonance imaging (MRI), which are often constrained by subjective interpretation, inter-observer variability, and reduced sensitivity in dense breast tissue. These limitations have driven a profound technological shift toward artificial intelligence (AI) and radiomics, enabling the high-throughput extraction of quantitative imaging features to non-invasively capture complex tumor heterogeneity. Crucially, this evolution has expanded beyond macroscopic radiological imaging into the frontier of AI-augmented pathology, or pathomics. By embracing multi-dimensional data integration—fusing radiomics, pathomics, and multi-omics—the field is achieving unprecedented precision in clinical oncology, shifting away from generalized guidelines toward individualized, dynamics-aware predictive modelling.

## 2 Modalities and algorithmic frameworks

The current landscape of breast cancer research leverages a broad spectrum of AI architectures to process diverse data modalities. For classical machine learning tasks handling tabular clinical data or extracted radiomic features, ensemble algorithms such as Random Forest (RF), Support Vector Machines (SVM), XGBoost, LightGBM, and CatBoost are predominantly utilized. Time-to-event prognosis frequently relies on specialized survival models, including the Accelerated Oblique Random Survival Forest (AORSF) (Wang et al.). Feature selection is rigorously managed via the Least Absolute Shrinkage and Selection Operator (LASSO) to minimize multicollinearity in high-dimensional datasets.

In the realm of deep learning, convolutional neural networks (CNNs), such as ResNet50, DenseNet, and specialized variants (ULNet, PLNet) drive transfer learning and automated image classification tasks (Cossio et al.; Peng et al.). Furthermore, advanced architectures—such as the Vision Transformer (ViT), the Unified Network for Image (UNI), the attentive tabular framework TabNet, and dynamics-aware sequence learners like ResponseNet—are increasingly deployed to capture global interactions and temporal patient treatment trajectories (Wang et al.; Wang et al.; Yue et al.). To establish a comprehensive macro-level overview of these algorithmic paradigms, task-aware bibliometric mapping across 608 peer-reviewed publications (2020–2026) confirms a decisive architectural shift from classical convolutional neural network (CNN) backbones toward Vision Transformers (ViTs) and hybrid attention mechanisms (Mohamed et al.). This quantitative synthesis demonstrates that while deep learning models increasingly dominate computational oncology, escalating model complexity underscores an urgent imperative for standardized hyperparameter tuning and transparent architectural reporting (Mohamed et al.).

The distribution of primary data modalities across the synthesized cohort is detailed in the table below (see **Table 1**):

**Table 1:** Distribution of primary data modalities along with a short description of the corresponding methodology across the synthesized cohort.

Modality/focus	Reference	Methodology & focus
Ultrasound	(Wang et al.)	Reviews AI-mediated ultrasound radiomics for extracting features in triple-negative breast cancer.
	(Wang et al.)	Uses Automated Breast Volume Scanning (ABVS) to predict neoadjuvant chemotherapy responses.
	(Peng et al.)	Proposes a multimodal fusion model utilizing preoperative ultrasound alongside histopathology
	(Xu et al.)	Extracts features from grayscale and wavelet-transformed ultrasound images to predict EGFR status.
	(Zilalan et al.)	Applies ultrasound radiomics to predict postoperative locoregional recurrence and distant metastasis.
MRI	(Ran et al.)	Reviews multi-omics integration and discusses multiparametric MRI radiomics for subtype differentiation.
Pathomics	(Cossio et al.)	Uses transfer learning on H&E-stained whole-slide images (WSIs) for sentinel lymph node metastasis detection.
	(Wang et al.)	Models patient-specific treatment trajectories using histopathological image classification.
	(Yue et al.)	Extracts pathomic features from digitized pretreatment biopsy slides to predict chemotherapy response.
	(Peng et al.)	Fuses histopathology core needle biopsy features with ultrasound data.
Multi-omics	(Fang et al.)	Constructs models using transcriptional multi-omics data focused on natural killer cell-related gene signatures.
	(Yan et al.)	Integrates single-cell regulatory atlases with bulk multi-omics to map responses to CDK4/6 inhibitors.
	(Ran et al.)	Reviews the application of AI in integrating multi-omics data for early diagnosis and drug resistance.
Other Modalities (Clinical, Genomic, Mammography)	(Guo et al.)	Focuses on Mammography images and radiomics to differentiate breast lesions.
	(Chen et al.)	Uses Clinical/SEER data to predict outcomes in breast cancer liver metastases.
	(Liu et al.)	Utilizes Epidemiological Global Burden of Disease (GBD) data to map global trends.
	(Wang et al.)	A systematic review of Clinical trial/RCT data assessing mindfulness and fatigue.
	(Wang et al.)	Uses Clinical/SEER data for external validation of genomic risk scores and guiding chemotherapy.
	(Zeng et al.)	Focuses on Molecular biology/RNA-seq data to evaluate CDK1 as a biomarker.
	(Li et al.)	Reviews Clinical genomics/Case report data regarding BRCA2 mutations.
	(Ballatore et al.)	Evaluates exceptional responses via Clinical case report data.
	(Yin et al.)	Focuses on Electronic Medical Records (EMR)/Clinical data to stratify thyroid cancer co-occurrence risk.
	(Zhang et al.)	Focuses on Theoretical nursing and Clinical workflow frameworks.
Systematic Review & Bibliometrics	(Mohamed et al.)	Systematically maps 608 publications (2020–2026) evaluating clinical tasks, XAI, HPO, and transformer architectures.

## 3 Overview of clinical themes

The integration of the aforementioned modalities delineates four core clinical sub-themes driving personalized oncology:

### 3.1 Early diagnosis & molecular profiling

AI and radiomics establish robust, non-invasive frameworks for differentiating tumor subtypes and identifying critical diagnostic signatures. For instance, mammographic radiomics combined with SVM models successfully distinguishes non-mass breast cancer from non-puerperal mastitis, addressing a major radiological challenge complicated by overlapping lesion architectures (Guo et al.). Similarly, ultrasound radiomics can non-invasively predict epidermal growth factor receptor (EGFR) expression status, uncovering crucial spatial tumor heterogeneity (Xu et al.). Furthermore, machine learning models incorporating natural killer (NK) cell-related gene signatures drawn from transcriptomic data successfully differentiate breast cancer tissues and stratify patient prognosis (Fang et al.). These multi-dimensional profiling tools are particularly beneficial in decoding highly invasive subtypes, such as triple-negative breast cancer (TNBC) (Ran et al.; Wang et al.).

### 3.2 Treatment response & resistance prediction

Accurately predicting therapeutic efficacy—especially to neoadjuvant chemotherapy (NAC)—is a pivotal step in avoiding ineffective treatments and associated toxicities. Pathomics-driven CatBoost models integrating digitized H&E slides and clinical parameters can reliably forecast NAC efficacy and early recurrence in HR-positive, HER2-negative patients (Yue et al.). Radiomics derived from Automated Breast Volume Scanning (ABVS) further optimizes NAC prediction by utilizing peritumoral features, demonstrating that the 6-mm peritumoral zone is highly predictive of response (Wang et al.). Crucially, a substantial task-level imbalance persists across the computational oncology literature. Quantitative analysis of a 608-paper corpus indicates that diagnostic applications, including detection, classification, and segmentation, comprise 71.2% of the published literature, whereas prognostic risk modeling accounts for 27.5% and therapeutic treatment-response forecasting represents only 2.3% (Mohamed et al.). This task-level imbalance underscores a vital opportunity to pivot research momentum from static lesion detection toward dynamic therapy response prediction and longitudinal risk stratification. Advanced sequence learning architectures simulate counterfactual patient trajectories to map personalized therapy responses over time (Wang et al.). AI also plays a critical role in decoding drug resistance; integrating single-cell regulatory atlases evaluates differential responses to CDK4/6 inhibitors via a Tumor Prognostic Regulon Index (TPRI) (Yan et al.). In the clinical domain, compelling case reports highlight exceptional responses to targeted agents like Trastuzumab deruxtecan (Ballatore et al.) and characterize multidrug primary resistance driven by germline BRCA2 exon 27 mutations (Li et al.). Finally, novel survival forest models effectively validate genomic risk panels like Oncotype DX to accurately guide adjuvant chemotherapy (Wang et al.).

### 3.3 Prognostic risk assessment

Prognostic models are essential for mapping the likelihood of locoregional recurrence and metastasis. Multimodal deep learning architectures that fuse preoperative ultrasound and core-needle biopsy images offer superior prediction of axillary lymph node metastasis compared to single-modality approaches (Peng et al.). Concurrently, AI-augmented pathology employing transfer learning on whole-slide images automates sentinel lymph node analysis with clinical-grade accuracy, demonstrating high resilience to interhospital domain shifts (Cossio et al.). For patients with TNBC, AI-driven ultrasound radiomics provides a powerful tool to predict postoperative locoregional recurrence and distinct patterns of distant metastasis (Zilalan et al.). Machine learning algorithms concurrently identify high-risk prognostic survival metrics for severe conditions such as breast cancer liver metastases (Chen et al.) and utilize XGBoost models to predict the co-occurrence of primary thyroid cancer (Yin et al.). From an epidemiological and molecular standpoint, statistical modeling maps the rising global burden of elderly male breast cancer (Liu et al.), while integrated transcriptomics identifies oncogenic drivers like CDK1 promoting progression through AKT activation (Zeng et al.).

### 3.4 Patient-centered care

The successful clinical translation of AI necessitates deep integration with humanistic, patient-centered care models. A comprehensive nursing-centered analytical framework asserts that while AI optimally handles data-heavy triage, decision support, and monitoring tasks, nurses must safeguard professional autonomy, preserve empathetic therapeutic relationships, and manage ethical governance across the personalized care continuum (Zhang et al.). In addressing holistic symptom management, a rigorous meta-analysis confirms that mindfulness-based stress reduction (MBSR) significantly mitigates cancer-related fatigue, improving patients’ overall quality of life and sleep continuity during therapy (Wang et al.).

## 4 Emerging trends and future challenges

The continuous evolution of computational oncology has accelerated the development of generative AI applications and dynamic decision-making frameworks for sequence exploration in drug design. However, the translation of AI into routine clinical workflows faces substantial challenges. Dataset generalizability is a primary concern; models trained on single-center, retrospective datasets frequently experience degraded performance when transitioning to diverse, real-world populations due to domain shifts. Critical methodological hurdles continue to impede the seamless translation of AI algorithms into routine clinical workflows. Although Explainable AI (XAI) visualizers—such as Grad-CAM, SHAP, and LIME—are increasingly deployed to mitigate black-box opacity, hyperparameter optimization (HPO) remains insufficiently reported, with only 15–25% of studies explicitly documenting tuning protocols (Mohamed et al.). Furthermore, while some transformer-based and hybrid architectures report near-perfect benchmark performance (98–100%), these metrics often stem from single-center retrospective cohorts prone to internal validation bias, reinforcing the necessity for prospective, multi-institutional external validation (Mohamed et al.). To build clinical trust, the pervasive “black box” problem of algorithmic interpretability must be mitigated. Studies are actively addressing this by developing intuitive visual interfaces, high-contrast probability heatmaps, and SHapley Additive exPlanations (SHAP) to provide feature attributions, allowing pathologists and oncologists to interact transparently with the model’s rationale. Ultimately, establishing the synergistic integration of AI into clinical environments relies on robust institutional governance, clearly defining the collaborative division of labor, override thresholds, and accountability between intelligent tools and human healthcare professionals.

## 5 Conclusion

The integration of artificial intelligence, advanced radiomics, and multi-omics represents a transformative milestone in the pursuit of precision medicine for breast cancer. By migrating from static diagnostic criteria to dynamic, patient-specific predictive frameworks, computational oncology provides highly actionable insights into early disease classification, therapeutic response forecasting, and longitudinal prognostic risk assessment. As researchers proactively confront the challenges of algorithmic transparency, dataset generalizability, and clinical workflow integration, the synergistic collaboration between human clinical expertise and AI-augmented analytics is poised to establish a new gold standard in personalized, patient-centered oncological care.

